# Supplementary material for: Climate change and Land Use/Land Cover Change (LUCC) leading to spatial shifts in net primary productivity in Anhui Province, China
Source: PLoS One. 2024 Sep 6;19(9):e0307516. doi: 10.1371/journal.pone.0307516 (PMC11379229; doi:10.1371/journal.pone.0307516)
Supplement: Attachment — Submitted filename: Response to Reviewers.docx [file pone.0307516.s001.docx]

**Response to reviewer comments**

(Manuscript Ref. No. PONE-D-24-16627)

Dear Editors,

I hope you are doing very well. The comments of the reviewer are very much appreciated and helped improve the manuscript significantly. We responded to your comments and made all the requested changes. Those changes are highlighted with red color in the revised manuscript with changes marked. In the following section, we explained in detail how we responded to each of the comments.

At first, the paper has been updated using the correct PONE template following the Guidelines.

**Reviewer #1:** Based on NPP data, climate data, and LUCC data, this study analyzed the spatiotemporal variation characteristics of NPP and its response to climate change in Anhui Province. The research results may contribute to further understand the relationship between NPP change and climate change. However, there are some concerns that the authors should address before it can be considered for publication.

1. Lines 32-34, I suggest the authors add more relevant references to back "It is the main factor to determine the carbon source and sink of the ecosystem and regulate the ecological process and is one of the important contents in the study of the carbon cycle process of the terrestrial ecosystem.

**Response:** Thank you for the positive and valuable comments. More relevant references are added to support “It is the main factor to determine the carbon source and sink of the ecosystem and regulate the ecological process and is one of the important contents in the study of the carbon cycle process of the terrestrial ecosystem”.

**After revision:**

**Line 32 to 34:**

Net Primary Productivity (NPP) refers to the energy remaining for plant growth, development and reproduction after the total organic matter synthesized by photosynthesis is subtracted from organic matter consumed by respiration. It is used to represent the carbon sequestration number of green plants [1,2]. NPP is the net photosynthetic production produced under the comprehensive action of the surrounding environment and the specific physiological characteristics of plants [3-5]. It is the main factor to determine the carbon source and sink of the ecosystem and regulate the ecological process and is one of the important contents in the study of the carbon cycle process of the terrestrial ecosystem [6-11]. The intensification and spatial expansion of human activities in recent centuries has profoundly altered the world's natural and cultural landscape and has had a significant impact on ecosystem processes and their social functioning [12]. A comprehensive indicator of this global change is the mechanism of change in vegetation productivity.

**Added references:**

3.	Cramer W, Kicklighter D, Bondeau A, Iii BM, Churkina G, Nemry B, et al. Comparing global models of terrestrial net primary productivity (NPP): overview and key results. Global Change Biology. 1999;5:iii–iv. doi: 10.1046/j.1365-2486.1999.00001.x.

4.	Field C, Behrenfeld M, Randerson J, Falkowski P. Primary production of the biosphere: Integrating terrestrial and oceanic components. Science 281: 237-240. Science (New York, NY). 1998;281:237-240. doi: 10.1126/science.281.5374.237.

5.	Xiaobin G, Huanfeng S, Wenxia G, Gang Y, Lunche W, Xinghua L, et al. A 33-Year NPP Monitoring Study in Southwest China by the Fusion of Multi-Source Remote Sensing and Station Data. Remote Sensing. 2017;9:1082-1101. doi: 10.3390/rs9101082.

6.	Nayak RK, Patel NR, Dadhwal VKJEM, Assessment. Estimation and analysis of terrestrial net primary productivity over India by remote-sensing-driven terrestrial biosphere model. Environmental Monitoring and Assessment. 2009;170:195-213. doi: 10.1007/s10661-009-1226-9.

7.	Zhao M, Running S. Drought-Induced Reduction in Global Terrestrial Net Primary Production from 2000 Through 2009. Science (New York, NY). 2010;329:940-943. doi: 10.1126/science.1192666.

8.	Pachavo G, Murwira AJGI. Land-use and land tenure explain spatial and temporal patterns in terrestrial net primary productivity (NPP) in Southern Africa. Geocarto International. 2014;29:671-687. doi: 10.1080/10106049.2013.837101.

9.	Piao S, Sitch S, Ciais P, Friedlingstein P, Peylin P, Wang X, et al. Evaluation of terrestrial carbon cycle models for their response to climate variability and to CO2 trends. Global Change Biology. 2013;19:2117–2132. doi: 10.1111/gcb.12187.

10.	Reyer C, Lasch P, Suckow F, Gutsch M, Murawski A, Pilz T. Projections of regional changes in forest net primary productivity for different tree species in Europe driven by climate change and carbon dioxide. Annals of Forest Science. 2014;71:211–225. doi: 10.1007/s13595-013-0306-8.

11.	Liang W, Yang Y, Fan D, Guan H, Zhang T, Long D, et al. Analysis of spatial and temporal patterns of net primary production and their climate controls in China from 1982 to 2010. Agricultural and Forest Meteorology. 2015;204:22-36. doi: 10.1016/j.agrformet.2015.01.015.

2. Where are the sources of data for descriptions of meteorological environmental factors in the study area?

**Response:** Thanks to the reviewer for highlighting our unique aspects. The sources of data for descriptions of meteorological environmental factors in the study area listed in Table 1 are added.

**After revision:**

**Line 106 to 106:**

**Table 1. Data sources.**

| **Data** | **Spatial resolution** | **Sources** |
| --- | --- | --- |
| Vector boundary data of China and Anhui Province | vector | https://www.webmap.cn/commres.do?method=result100W |
| MOD13Q1 NDVI | 250m×250m | https://ladsweb.modaps.eosdis.nasa.gov/search/ |
| monthly mean temperature | - | https://www.resdc.cn/data.aspx?DATAID=349, GST_ave |
| monthly total solar radiation | - | https://www.resdc.cn/data.aspx?DATAID=349, SSD |
| monthly total precipitation | - | https://www.resdc.cn/data.aspx?DATAID=349, PRE |
| Vegetation type | 1km×1km | [https://www.plantplus.cn/doi/10.12282/plantdata.0155](https://www.plantplus.cn/doi/10.12282/plantdata.0155%20) |
| land-use and land-cover | 30m×30m | https://www.webmap.cn/mapDataAction.do?method=globalLandCover |

**Line 115 to 122:**

(3) Meteorological data. The climate dataset was derived from 77 climatological stations (Fig 1(b)) in Anhui province, including three types of data from 2001 to 2020: temperature, precipitation, and solar radiation, and were provided by Resource and Environmental Science Data Platform (https://www.resdc.cn/data.aspx?DATAID=349). To ensure the quality of data, we delete the suspicious and missing records. Since many researches have demonstrated that the Kriging method is an effective interpolation method, having been widely used for regionalizing various variables at different scales [44-45], these climatological data were interpolated through the Kriging method, to produce raster images. All data were resampled at a resolution of 250 m× 250 m and repeatedly projected for WGS_1984_Albers. Time range consistent with NDVI.

**Added references:**

44.	Zhu W, Pan Y, He H, Yu D, Hu H. Simulation of maximum light use efficiency for some typical vegetation types in China. Chinese Science Bulletin. 2006;51:457-463. doi: 10.1007/s11434-006-0457-1.

45.	Liu C, Dong X, Liu YJC. Changes of NPP and their relationship to climate factors based on the transformation of different scales in Gansu, China. Catena 2015;125:190-199. doi: 10.1016/J.CATENA.2014.10.027.

3. How do the authors account for missing values in meteorological data?

**Response:** Thanks to the reviewer for your valuable comments. We obtained data from 77 stations from the Resource and Environmental Science Data Platform, and there are very few missing values every year. To ensure the quality of data, we delete the suspicious and missing records.

**After revision:**

**Line 115 to 122:**

(3) Meteorological data. The climate dataset was derived from 77 climatological stations (Fig 1(b)) in Anhui province, including three types of data from 2001 to 2020: temperature, precipitation, and solar radiation, and were provided by Resource and Environmental Science Data Platform (https://www.resdc.cn/data.aspx?DATAID=349). To ensure the quality of data, we delete the suspicious and missing records. Since many researches have demonstrated that the Kriging method is an effective interpolation method, having been widely used for regionalizing various variables at different scales [44-45], these climatological data were interpolated through the Kriging method, to produce raster images. All data were resampled at a resolution of 250 m× 250 m and repeatedly projected for WGS_1984_Albers. Time range consistent with NDVI.

4. I suggest the authors add a diagram about the spatial distribution of meteorological stations in Anhui Province.

**Response:** Thanks to the reviewer for your valuable comments. A diagram about the spatial distribution of meteorological stations in Anhui Province has been added to figure 1(b). The red dots in the picture are the meteorological stations.

**After revision:**

**Line 102 to 102:**

**Fig 1. Geographical location of the study area.**

**Line 115 to 122:**

(3) Meteorological data. The climate dataset was derived from 77 climatological stations (Fig 1(b)) in Anhui province, including three types of data from 2001 to 2020: temperature, precipitation, and solar radiation, and were provided by Resource and Environmental Science Data Platform (https://www.resdc.cn/data.aspx?DATAID=349). To ensure the quality of data, we delete the suspicious and missing records. Since many researches have demonstrated that the Kriging method is an effective interpolation method, having been widely used for regionalizing various variables at different scales [44-45], these climatological data were interpolated through the Kriging method, to produce raster images. All data were resampled at a resolution of 250 m× 250 m and repeatedly projected for WGS_1984_Albers. Time range consistent with NDVI.

5. How does the authors account for the inconsistency of spatial resolution between different data sets?

**Response:** Thanks to the reviewer for your valuable comments. All data were resampled at a resolution of 250 m × 250 m and repeatedly projected for WGS_1984_Albers.

**After revision:**

**Line 115 to 122:**

(3) Meteorological data. The climate dataset was derived from 77 climatological stations (Fig 1(b)) in Anhui province, including three types of data from 2001 to 2020: temperature, precipitation, and solar radiation, and were provided by Resource and Environmental Science Data Platform (https://www.resdc.cn/data.aspx?DATAID=349). To ensure the quality of data, we delete the suspicious and missing records. Since many researches have demonstrated that the Kriging method is an effective interpolation method, having been widely used for regionalizing various variables at different scales [44-45], these climatological data were interpolated through the Kriging method, to produce raster images. All data were resampled at a resolution of 250 m× 250 m and repeatedly projected for WGS_1984_Albers. Time range consistent with NDVI.

**Line 127 to 131:**

(5) land-use and land-cover (LULC). The remote sensing data with a resolution of 30m*30m was provided by national catalogue service for geographic information (https://www.webmap.cn/mapDataAction.do?method=globalLandCover). We resampled it to a resolution of 250 meters using the nearest neighbor method based on ArcGIS 10.8 [45]. Land use types include divided into Cultivated Land, Forest, Grassland, Wetland, Water, Artificial Surfaces and Bare land.

6. More mechanistic explanations should be added to further explain the relationship between NPP and climate factors.

**Response:** Thanks to the reviewer for your valuable comments. We have added partial correlation analysis between NPP and climate factors on the basis of simple correlation analysis.

**After revision:**

**Line 375 to 425:**

Partial correlation analysis of monthly scale NPP and meteorological factors

On the basis of correlation analysis, we further conduct partial correlation analysis. Fig 11 (a) displayed the partial correlation distribution between NPP and temperature. The results show that after fixed precipitation and solar radiation, the average partial correlation coefficient between NPP and temperature is 0.03, range from -0.89 to 0.84. The area of the region that reached a significant level (P<0.05) only accounts for 4.34% of Anhui Province, and 44.99% of the regions showed a negative correlation. The partial correlation distribution between NPP and precipitation is presented in Fig 11 (b). The average partial correlation coefficient is 0.26, and the area that reaches a significant level (P<0.05) accounts for 13.35% of the total area. After ignoring the effects of temperature and precipitation, the average partial correlation coefficient between NPP and solar radiation is 0.47, and the area ratio that reaches a significant level (P<0.05) is 37.42% (Fig 11 (c)).

Previous studies have shown that warming and wetting of climate can promote vegetation growth [1,69,75]. However, after eliminating the effects of precipitation and solar radiation, this article found that the correlation between NPP and temperature rapidly decreased, and the area that reached a significant level also rapidly decreased. Some areas also showed a negative correlation, mainly distributed on cultivated land on both sides of the Yangtze and Huaihe Rivers. Rice is planted on both sides of the Yangtze River, and wheat is planted on both sides of the Huaihe River. High temperatures can inhibit the growth of two types of crops, which rely more on water and light hours. Du et al. [55] reached a similar conclusion when studying the partial correlation analysis between NDVI and temperature, which is that there is a negative correlation in the western and southern parts of Anhui Province. After eliminating the effects of temperature and solar radiation, the correlation between precipitation and NPP has also weakened, with negative correlations appearing on both sides of the Yangtze River and around lakes where soil moisture is abundant and excessive precipitation can inhibit vegetation growth. After eliminating the effects of temperature and precipitation, the correlation between solar radiation and NPP slightly decreased. Similar to the results of simple correlation analysis, except for water, all other regions showed a positive correlation. The explanation is that any unilateral change in climate factors is not sufficient to cause significant changes in NPP of large-scale pixel groups, but rather the result of the coupling effect of heat, water, and energy.

The response of NPP to climate factors varies among different land cover types. The partial correlation coefficients between cultivated land and temperature, precipitation, and solar radiation are 0.03, 0.29, and 0.46, respectively, with some showing a negative correlation. This may be because some crops in Anhui Province that are suitable for low-temperature growth are limited in carbon accumulation during high temperatures, while sufficient rainfall and longer sunshine hours result in more carbon accumulation. Especially in the Jianghuai region, it is greatly affected by precipitation, due to the fact that the vegetation in this area is mainly dry land. In forested areas, the partial correlation coefficients of temperature, precipitation, and solar radiation are 0.01, 0.21, and 0.55, respectively. The forest land in Anhui Province is located in relatively high altitude mountainous areas, with a large distribution of warm and cool vegetation and strong drought resistance. The longer the sunshine hours, the more favorable it is for carbon accumulation. This is similar to Du et al. [56], who mentioned in his article that the partial correlation coefficient between forest NPP in Anhui Province and temperature and precipitation is around 0, while the partial correlation coefficient with solar radiation is around 0.45. The partial correlation coefficients between grassland and temperature, precipitation, and solar radiation are 0.03, 0.29, and 0.48, respectively. The partial correlation coefficients between cities and temperature, precipitation, and solar radiation are 0.03, 0.31, and 0.42, respectively Relatively speaking, vegetation in grasslands and cities has a higher dependence on precipitation than other land cover types. Increasing precipitation can promote vegetation growth and increase the annual NPP of grasslands by increasing soil moisture in the region [76]. The more precipitation, the longer the sunshine hours, which are beneficial for carbon accumulation. Among other land cover types, the proportion of correlation with NPP reaching a significant level is relatively small. Overall, through partial correlation analysis, it was found that solar radiation has the greatest impact on the NPP of Anhui Province, followed by precipitation, and the least impact on temperature.

(a) partial correlation analysis between NPP and temperature.

(b) partial correlation analysis between NPP and precipitation.

(c) partial correlation analysis between NPP and solar radiation.

Fig 11. Spatial distribution of the partial correlation analysis between NPP and meteorological factors.

**Added references:**

55.	Du Z, Zhao J, Liu X, Wu Z, Zhang HJES, Research P. Recent asymmetric warming trends of daytime versus nighttime and their linkages with vegetation greenness in temperate China. Environmental Science and Pollution Research. 2019;26:35717-35727. doi: 10.1007/s11356-019-06440-z.

56.	Du Z, Liu X, Wu Z, Zhang H, Zhao J. Responses of Forest Net Primary Productivity to Climatic Factors in China during 1982–2015. Plants. 2022;11:2932-2949. doi: 10.3390/plants11212932.

76.	Guo D, Song X, Hu R, Cai S, Zhu X, Hao Y. Grassland type-dependent spatiotemporal characteristics of productivity in Inner Mongolia and its response to climate factors. Science of The Total Environment. 2021;775:145644-53. doi: 10.1016/j.scitotenv.2021.145644.

7. In the uncertainty, I suggest the authors further discuss the uncertainty of remote sensing data including NPP data (e.g., Shen et al., 2022; Ma et al., 2022) which may affect the research results.

**Response:** Thanks to the reviewer for your valuable comments. We have added uncertainty analysis in the discussion section.

**After revision:**

**Line 464 to 474:**

Uncertainty

Although this study provided a comprehensive analysis of NPP changes and climate effects in Anhui Province, there may still be some limitations. Firstly, there are deficiencies in the accuracy verification of the CASA model. Due to the inability of local relevant departments to provide on-site measurement data, this article only compares it with existing research data and MOD17A3 data. This is also one of the problems that many scholars face in studying NPP, which may cause some uncertainty [77]. This article uses NDVI and meteorological data to calculate NPP, NPP data could contain some inaccuracies which may affect the results of this study. The interpolation method of meteorological data may also affect the results of NPP [50,52]. In addition, this article analyzes the effects of temperature, precipitation, solar radiation and urbanization on NPP, though there are some other factors (such as humidity, LUCC, etc.) that can also affect NPP. Future studies are still needed to further explore the impacts of temperature and precipitation on NPP and the effects of other climatic factors on NPP in Anhui Province.

**Added references:**

50.	Ma R, Xia C, Liu Y, Wang Y, Zhang J, Shen X, et al. Spatiotemporal Change of Net Primary Productivity and Its Response to Climate Change in Temperate Grasslands of China. Frontiers in Plant Science. 2022; 13:899800-899811. doi: 10.3389/fpls.2022.899800.

52.	Shen X, Liu Y, Zhang J, Wang Y, Ma R, Liu B, et al. Asymmetric Impacts of Diurnal Warming on Vegetation Carbon Sequestration of Marshes in the Qinghai Tibet Plateau. Global Biogeochemical Cycles. 2022;36: e2022GB007396- e2022GB007409. doi: 10.1029/2022GB007396.

77.	Shao S, Yang Y. Analysis of change process of NPP dominated by human activities in Northwest Hubei, China, from 2000 to 2020. Environmental Science and Pollution Research. 2024; 31:1-13. doi: 10.1007/s11356-024-32370-6.

8. In order to further highlight the innovation of this article, it is better to compare the results of this study with some related studies.

**Response:** Thanks to the reviewer for your valuable comments. We have added comparison with relevant research.

**After revision:**

**Line 278 to 285:**

Forests in Anhui province are mainly concentrated in the Dabie Mountains and the Jiangnan Mountains. The average NPP is 603.17 gC·(m2 ·a) -1. Research by Sun et al. [32] and Wang et al. [2] shows that In southern China, the evergreen broad-leaved forest is widely distributed and rich in resources, the annual average NPP is higher than 600 gC·(m2 ·a) -1. The values between 400 and 700 gC·(m2 ·a) -1 were located in the middle and lower reaches of the Yangtze River. Rich precipitation and groundwater are more conducive to the growth of vegetation. It is consistent with the conclusion of this paper. The NPP of forest land in this article has shown an upward trend over the past two decades. The upward trend of forest NPP is consistent with the increase of land vegetation NPP in China and the Northern Hemisphere [11,56].

**Line 340 to 346:**

Fang et al. [69] indicated that temperature is the main factor controlling changes in NPP in humid and semi-humid areas. Anhui Province belongs to the humid areas of warm temperate and subtropical regions, we also found that temperature has the highest impact on NPP, followed by solar radiation, and finally precipitation. A warm and humid climate, sufficient precipitation and heat are conducive to the increase of vegetation NPP in Anhui Province [70-71]. Just like in most regions of the Northern Hemisphere, as temperatures rise, vegetation enters the growing season earlier and grows longer, which enhances photosynthesis and promotes vegetation coverage [72-73].

**Line 365 to 370:**

The monthly mean NPP of different land cover types also has different feedback on monthly scale climate factors such as temperature, precipitation, and solar radiation. In Table 4, among the relationship between NPP and temperature, forest land has the greatest correlation, reaching 0.85 (P<0.1). And then the grass, Cultivated Land (0.72) > Artificial Surfaces (0.63) > Wetland \ Bare land (0.59) > Water (0.50). In the relationship between NPP and precipitation, the correlation of grassland was 0.47 (P<0.1). The correlation coefficient of cultivated land was 0.46. The correlation coefficient of other land cover types was less than 0.40. In the relationship between NPP and solar radiation, the correlation of forest land is still the largest, reaching 0.69(P<0.1). It was followed by grassland with correlation coefficient of 0.60. Then it was cultivated land > Wetland > Artificial Surfaces > Bare land > Water. And the correlation with temperature is highly consistent. It shows that temperature and solar radiation have a high degree of consistency. In general, the effect of monthly mean temperature on NPP of all land cover types was greater than that of monthly precipitation, especially for forest land and grassland. It may be because the water system in Anhui province is developed, and the carbon sequestration capacity of vegetation is more affected by temperature. Zhang et al. [74] showed in his paper that the correlation between air temperature and forest NPP in the Yangtze River Basin was stronger than that between precipitation and forest NPP, which was consistent with the results of this paper. Our results showed that precipitation had a lower correlation with forest NPP than temperature and solar radiation, this is in agreement with Du et al. [56], which indicating that precipitation has a smaller impact on forest vegetation growth than temperature and solar radiation. Our findings even showed that a positive correlation between grassland NPP and precipitation. This is in agreement with Ma et al. [50], who found that the increased precipitation could significantly increase the annual NPP of temperate grasslands.

**Line 384 to 420:**

Previous studies have shown that warming and wetting of climate can promote vegetation growth [1,69,75]. However, after eliminating the effects of precipitation and solar radiation, this article found that the correlation between NPP and temperature rapidly decreased, and the area that reached a significant level also rapidly decreased. Some areas also showed a negative correlation, mainly distributed on cultivated land on both sides of the Yangtze and Huaihe Rivers. Rice is planted on both sides of the Yangtze River, and wheat is planted on both sides of the Huaihe River. High temperatures can inhibit the growth of two types of crops, which rely more on water and light hours. Du et al. [55] reached a similar conclusion when studying the partial correlation analysis between NDVI and temperature, which is that there is a negative correlation in the western and southern parts of Anhui Province. After eliminating the effects of temperature and solar radiation, the correlation between precipitation and NPP has also weakened, with negative correlations appearing on both sides of the Yangtze River and around lakes where soil moisture is abundant and excessive precipitation can inhibit vegetation growth. After eliminating the effects of temperature and precipitation, the correlation be-tween solar radiation and NPP slightly decreased. Similar to the results of simple correlation analysis, except for water, all other regions showed a positive correlation. The explanation is that any unilateral change in climate factors is not sufficient to cause significant changes in NPP of large-scale pixel groups, but rather the result of the coupling effect of heat, water, and energy.

The response of NPP to climate factors varies among different land cover types. The partial correlation coefficients between cultivated land and temperature, precipitation, and solar radiation are 0.03, 0.29, and 0.46, respectively, with some showing a negative correlation. This may be because some crops in Anhui Province that are suitable for low-temperature growth are limited in carbon accumulation during high temperatures, while sufficient rainfall and longer sunshine hours result in more carbon accumulation. Especially in the Jianghuai region, it is greatly affected by precipitation, due to the fact that the vegetation in this area is mainly dry land. In forested areas, the partial correlation coefficients of temperature, precipitation, and solar radiation are 0.01, 0.21, and 0.55, respectively. The forest land in Anhui Province is located in relatively high altitude mountainous areas, with a large distribution of warm and cool vegetation and strong drought resistance. The longer the sunshine hours, the more favorable it is for carbon accumulation. This is similar to Du et al. [56], who men-tioned in his article that the partial correlation coefficient between forest NPP in Anhui Province and temper-ature and precipitation is around 0, while the partial correlation coefficient with solar radiation is around 0.45. The partial correlation coefficients between grassland and temperature, precipitation, and solar radiation are 0.03, 0.29, and 0.48, respectively. The partial correlation coefficients between cities and temperature, precipita-tion, and solar radiation are 0.03, 0.31, and 0.42, respectively Relatively speaking, vegetation in grasslands and cities has a higher dependence on precipitation than other land cover types. Increasing precipitation can pro-mote vegetation growth and increase the annual NPP of grasslands by increasing soil moisture in the region [76]. The more precipitation, the longer the sunshine hours, which are beneficial for carbon accumulation. Among other land cover types, the proportion of correlation with NPP reaching a significant level is relatively small. Overall, through partial correlation analysis, it was found that solar radiation has the greatest impact on the NPP of Anhui Province, followed by precipitation, and the least impact on temperature.

**Line 426 to 443:**

Land use and land cover change (LUCC) will directly affect the types, structures, and functions of ecosystems, and then have important impact on NPP. The most important land cover changes in Anhui Province from 2001 to 2020 was cultivated land to artificial surfaces, as well as the transformation between cultivated land and forest (See Table 5). Among all land cover types, the average annual NPP decreased most obviously, followed by bare land, and the average annual NPP increased for other land cover types. In order to understand the impact of land use change on NPP, we focused on studying the transformation from various land use types to artificial surfaces [28]. The area of artificial surfaces has increased by 4555.71 km^2^ in the past two decades. From Table 5, it can be seen that a total of 7377.80 km^2^ of cultivated land was converted into artificial surfaces, 406.27 km^2^ of forest has been converted into artificial surfaces, and 122.69 km^2^ of grassland has been converted into artificial surfaces. The unit area NPP of cultivated land, forest, and grassland are larger than that of artificial surfaces, so this transformation will cause losses in NPP. At the same time, water, wetlands, and bare land are converted into artificial surfaces, and their unit area NPP are smaller than that of artificial surfaces, which can lead to an increase in NPP. The urban expansion in Anhui Province is mainly derived from the conversion of cultivated land, so the urban expansion in Anhui Province has led to a decrease in NPP. The conclusion of Wang et al. [33] also verified the significant negative effect of urbanization on the productivity of vegetation cover system in Anhui province. Urban sprawl offsets the increase in NPP caused by climate change. The results of Yang et al. also indicate that the changes of NPP in the Yangtze River Basin are jointly influenced by LUCC changes and climate changes [60,75].

**Added references:**

1.	Wei X, Yang J, Luo P, Lin L, Lin K, Guan J. Assessment of the variation and influencing factors of vegetation NPP and carbon sink capacity under different natural conditions. Ecological Indicators. 2022;138:108834-108849. doi: 10.1016/j.ecolind.2022.108834.

11.	Liang W, Yang Y, Fan D, Guan H, Zhang T, Long D, et al. Analysis of spatial and temporal patterns of net primary production and their climate controls in China from 1982 to 2010. Agricultural and Forest Meteorology. 2015;204:22-36. doi: 10.1016/j.agrformet.2015.01.015.

28.	Li J, Wang Z, Lai C, Wu X, Zeng Z, Chen X, et al. Response of net primary production to land use and land cover change in mainland China since the late 1980s. The Science of the total environment. 2018;639:237-247. doi: 10.1016/j.scitotenv.2018.05.155.

50.	Ma R, Xia C, Liu Y, Wang Y, Zhang J, Shen X, et al. Spatiotemporal Change of Net Primary Productivity and Its Response to Climate Change in Temperate Grasslands of China. Frontiers in Plant Science. 2022;13:899800-899811. doi: 10.3389/fpls.2022.899800.

55.	Du Z, Zhao J, Liu X, Wu Z, Zhang HJES, Research P. Recent asymmetric warming trends of daytime versus nighttime and their linkages with vegetation greenness in temperate China. Environmental Science and Pollution Research. 2019;26:35717-35727. doi: 10.1007/s11356-019-06440-z.

56.	Du Z, Liu X, Wu Z, Zhang H, Zhao J. Responses of Forest Net Primary Productivity to Climatic Factors in China during 1982–2015. Plants. 2022;11:2932-2949. doi: 10.3390/plants11212932.

69.	Fang X, Zhang C, Wang Q, Chen X, Ding J, Karamage F. Isolating and Quantifying the Effects of Climate and CO2 Changes (1980–2014) on the Net Primary Productivity in Arid and Semiarid China. Forests. 2017;8:60-79. doi: 10.3390/f8030060.

70.	Fu Y, Lu X, Zhao Y, Zeng X, Xia L. Assessment Impacts of Weather and Land Use/Land Cover (LULC) Change on Urban Vegetation Net Primary Productivity (NPP): A Case Study in Guangzhou, China. Remote Sensing. 2013;5:4125-4144. doi: 10.3390/rs5084125. PubMed PMID: doi:10.3390/rs5084125.

71.	Pei F, Li X, Liu X, Lao C, Xia G. Exploring the response of net primary productivity variations to urban expansion and climate change: A scenario analysis for Guangdong Province in China. Journal of Environmental Management. 2015;150:92-102. doi: 10.1016/j.jenvman.2014.11.002.

72.	Shen M, Cong N, Cao R. Temperature sensitivity as an explanation of the latitudinal pattern of green-up date trend in Northern Hemisphere vegetation during 1982-2008. International Journal of Climatology. 2014;35:3707-3712. doi: 10.1002/joc.4227.

73.	Xiong L, Bai X, Zhao C, Li Y, Tan Q, Yun L, et al. High‐Resolution Data Sets for Global Carbonate and Silicate Rock Weathering Carbon Sinks and Their Change Trends. Earth's Future. 2022;10:e2022EF002746- e2022EF002763. doi: 10.1029/2022EF002746.

75.	Yang H, Hu D, Xu H, Zhong X. Assessing the spatiotemporal variation of NPP and its response to driving factors in Anhui province, China. Environmental Science and Pollution Research. 2020;27: 14915-14932. doi: 10.1007/s11356-020-08006-w.

76.	Guo D, Song X, Hu R, Cai S, Zhu X, Hao Y. Grassland type-dependent spatiotemporal characteristics of productivity in Inner Mongolia and its response to climate factors. Science of The Total Environment. 2021;775:145644-53. doi: 10.1016/j.scitotenv.2021.145644.

9. Why does the authors not consider the partial correlation method to measure the correlation between meteorological factors and NPP?

**Response:** Thanks to the reviewer for your valuable comments. We have added partial correlation analysis between NPP and climate factors on the basis of simple correlation analysis.

**After revision:**

**Line 375 to 424:**

Partial correlation analysis of monthly scale NPP and meteorological factors

On the basis of correlation analysis, we further conduct partial correlation analysis. Fig 11 (a) displayed the partial correlation distribution between NPP and temperature. The results show that after fixed precipitation and solar radiation, the average partial correlation coefficient between NPP and temperature is 0.03, range from -0.89 to 0.84. The area of the region that reached a significant level (P<0.05) only accounts for 4.34% of Anhui Province, and 44.99% of the regions showed a negative correlation. The partial correlation distribution between NPP and precipitation is presented in Fig 11 (b). The average partial correlation coefficient is 0.26, and the area that reaches a significant level (P<0.05) accounts for 13.35% of the total area. After ignoring the effects of temperature and precipitation, the average partial correlation coefficient between NPP and solar radiation is 0.47, and the area ratio that reaches a significant level (P<0.05) is 37.42% (Fig 11 (c)).

Previous studies have shown that warming and wetting of climate can promote vegetation growth [1,69,75]. However, after eliminating the effects of precipitation and solar radiation, this article found that the correlation between NPP and temperature rapidly decreased, and the area that reached a significant level also rapidly decreased. Some areas also showed a negative correlation, mainly distributed on cultivated land on both sides of the Yangtze and Huaihe Rivers. Rice is planted on both sides of the Yangtze River, and wheat is planted on both sides of the Huaihe River. High temperatures can inhibit the growth of two types of crops, which rely more on water and light hours. Du et al. [55] reached a similar conclusion when studying the partial correlation analysis between NDVI and temperature, which is that there is a negative correlation in the western and southern parts of Anhui Province. After eliminating the effects of temperature and solar radiation, the correlation between precipitation and NPP has also weakened, with negative correlations appearing on both sides of the Yangtze River and around lakes where soil moisture is abundant and excessive precipitation can inhibit vegetation growth. After eliminating the effects of temperature and precipitation, the correlation between solar radiation and NPP slightly decreased. Similar to the results of simple correlation analysis, except for water, all other regions showed a positive correlation. The explanation is that any unilateral change in climate factors is not sufficient to cause significant changes in NPP of large-scale pixel groups, but rather the result of the coupling effect of heat, water, and energy.

The response of NPP to climate factors varies among different land cover types. The partial correlation coefficients between cultivated land and temperature, precipitation, and solar radiation are 0.03, 0.29, and 0.46, respectively, with some showing a negative correlation. This may be because some crops in Anhui Province that are suitable for low-temperature growth are limited in carbon accumulation during high temperatures, while sufficient rainfall and longer sunshine hours result in more carbon accumulation. Especially in the Jianghuai region, it is greatly affected by precipitation, due to the fact that the vegetation in this area is mainly dry land. In forested areas, the partial correlation coefficients of temperature, precipitation, and solar radiation are 0.01, 0.21, and 0.55, respectively. The forest land in Anhui Province is located in relatively high altitude mountainous areas, with a large distribution of warm and cool vegetation and strong drought resistance. The longer the sunshine hours, the more favorable it is for carbon accumulation. This is similar to Du et al. [56], who mentioned in his article that the partial correlation coefficient between forest NPP in Anhui Province and temperature and precipitation is around 0, while the partial correlation coefficient with solar radiation is around 0.45. The partial correlation coefficients between grassland and temperature, precipitation, and solar radiation are 0.03, 0.29, and 0.48, respectively. The partial correlation coefficients between cities and temperature, precipitation, and solar radiation are 0.03, 0.31, and 0.42, respectively Relatively speaking, vegetation in grasslands and cities has a higher dependence on precipitation than other land cover types. Increasing precipitation can promote vegetation growth and increase the annual NPP of grasslands by increasing soil moisture in the region [76]. The more precipitation, the longer the sunshine hours, which are beneficial for carbon accumulation. Among other land cover types, the proportion of correlation with NPP reaching a significant level is relatively small. Overall, through partial correlation analysis, it was found that solar radiation has the greatest impact on the NPP of Anhui Province, followed by precipitation, and the least impact on temperature.

(a) partial correlation analysis between NPP and temperature.

(b) partial correlation analysis between NPP and precipitation.

(c) partial correlation analysis between NPP and solar radiation.

Fig 11. Spatial distribution of the partial correlation analysis between NPP and meteorological factors.

**Added references:**

55.	Du Z, Zhao J, Liu X, Wu Z, Zhang HJES, Research P. Recent asymmetric warming trends of daytime versus nighttime and their linkages with vegetation greenness in temperate China. Environmental Science and Pollution Research. 2019;26:35717-35727. doi: 10.1007/s11356-019-06440-z.

56.	Du Z, Liu X, Wu Z, Zhang H, Zhao J. Responses of Forest Net Primary Productivity to Climatic Factors in China during 1982–2015. Plants. 2022;11:2932-2949. doi: 10.3390/plants11212932.

76.	Guo D, Song X, Hu R, Cai S, Zhu X, Hao Y. Grassland type-dependent spatiotemporal characteristics of productivity in Inner Mongolia and its response to climate factors. Science of The Total Environment. 2021;775:145644-53. doi: 10.1016/j.scitotenv.2021.145644.

References:
Asymmetric impacts of diurnal warming on vegetation carbon sequestration of marshes in the Qinghai Tibet Plateau. Global Biogeochemical Cycles, 2022, 36(7): e2022GB007396.
Spatiotemporal change of net primary productivity and its response to climate change in temperate grasslands of China. Frontiers in Plant Science, 2022, 13: 899800.

**Reviewer #2:** Overall, this paper has a clear research framework and is a well-structured academic paper. However, I have a few main questions:

My biggest question is, since the author uses MODIS NPP data to validate the results of the CASA model, why not directly use the MODIS product?

**Response:** Thank you for the positive and valuable comments. Thanks to the reviewer for highlighting our unique aspects. MODIS NPP data is indeed widely used, but the artificial surfaces and water in this series of data are all null values. This article needs to use statistics and analysis to examine the NPP changes of various land use types, the impact of meteorological factors on NPP of different land use types, and the impact of land use changes on NPP. As a result, the CASA model was used to estimate NPP. We mainly used data from MODIS NPP on cultivated land, forest land, grassland, and bare land areas for validation, without selecting data from artificial surfaces and water areas.

**After revision:**

**Line 52 to 56:**

MOD17 is an NPP product of MODIS (Moderate-resolution Imaging Spectroradiometer), but the artificial surfaces and water in this series of data are all null values. This article needs to use statistics and analysis to examine the NPP changes of various land use types, the impact of meteorological factors on NPP of different land use types, and the impact of land use changes on NPP. However, using the CASA model, NPP for all land use types can be simulated. Therefore, this article uses the CASA model to estimate the NPP of Anhui Province.

Furthermore, there are many similar studies, even on national and global scales. What is the significance or innovation of this study? This needs to be highlighted in the introduction and fully referenced in the discussion section. For example:

Du, Z.; Liu, X.; Wu, Z.; Zhang, H.; Zhao, J. Responses of Forest Net Primary Productivity to Climatic Factors in China during 1982–2015. Plants 2022, 11, 2932. https://doi.org/10.3390/plants11212932;

Shang, E.; Xu, E.; Zhang, H.; Liu, F. Analysis of Spatiotemporal Dynamics of the Chinese Vegetation Net Primary Productivity from the 1960s to the 2000s. Remote Sens. 2018, 10, 860;

Li, J.; Wang, Z.; Lai, C.; Wu, X.; Zeng, Z.; Chen, X.; Lian, Y. Response of net primary production to land use and land cover change in mainland China since the late 1980s. Sci. Total Environ. 2018, 639, 237–247.

**Response:** Thank you for the positive and valuable comments. Thanks to the reviewer for highlighting our unique aspects. We have added innovation of this study in the introduction and fully referenced in the discussion section.

**After revision:**

**Line 81 to 91:**

Previous studies have focused more on the relationship between NPP and temperature precipitation, with limited analysis of the correlation between solar radiation; However, there is even less analysis of the correlation between NPP of different land use types and three meteorological factors. The unit area NPP varies among different land use types, so the transfer between different land use types can lead to changes in NPP. The increase in low-density carbon land use types and the decrease in high-density carbon land use types will lead to a decrease in NPP. On the contrary, NPP will increase. Therefore, this paper takes Anhui Province as the research object, using the improved CASA model and ArcGIS, ENVI and other software for research and application. Using 250m resolution MODIS NDVI (normalized difference vegetation index) data, vegetation type data, meteorological data (precipitation data, temperature data, total solar radiation) and land use data from 2001 to 2020, The analysis of driving factors of spatial heterogeneity of NPP changes in Anhui province was detailed, to judge the spatial-temporal pattern of NPP and the driving force of NPP influencing factors in Anhui province.

**Line 340 to 346:**

Fang et al. [69] indicated that temperature is the main factor controlling changes in NPP in humid and semi-humid areas. Anhui Province belongs to the humid areas of warm temperate and subtropical regions, we also found that temperature has the highest impact on NPP, followed by solar radiation, and finally precipitation. A warm and humid climate, sufficient precipitation and heat are conducive to the increase of vegetation NPP in Anhui Province [70-71]. Just like in most regions of the Northern Hemisphere, as temperatures rise, vegetation enters the growing season earlier and grows longer, which enhances photosynthesis and promotes vegetation coverage [72-73].

**Line 364 to 371:**

Zhang et al. [74] showed in his paper that the correlation between air temperature and forest NPP in the Yangtze River Basin was stronger than that between precipitation and forest NPP, which was consistent with the results of this paper. Our results showed that precipitation had a lower correlation with forest NPP than temperature and solar radiation, this is in agreement with Du et al. [56], which indicating that precipitation has a smaller impact on forest vegetation growth than temperature and solar radiation. Our findings even showed that a positive correlation between grassland NPP and precipitation. This is in agreement with Ma et al. [50], who found that the increased precipitation could significantly increase the annual NPP of temperate grass-lands.

**Line 375 to 425:**

Partial correlation analysis of monthly scale NPP and meteorological factors

On the basis of correlation analysis, we further conduct partial correlation analysis. Fig 11 (a) displayed the partial correlation distribution between NPP and temperature. The results show that after fixed precipitation and solar radiation, the average partial correlation coefficient between NPP and temperature is 0.03, range from -0.89 to 0.84. The area of the region that reached a significant level (P<0.05) only accounts for 4.34% of Anhui Province, and 44.99% of the regions showed a negative correlation. The partial correlation distribution between NPP and precipitation is presented in Fig 11 (b). The average partial correlation coefficient is 0.26, and the area that reaches a significant level (P<0.05) accounts for 13.35% of the total area. After ignoring the effects of temperature and precipitation, the average partial correlation coefficient between NPP and solar radiation is 0.47, and the area ratio that reaches a significant level (P<0.05) is 37.42% (Fig 11 (c)).

Previous studies have shown that warming and wetting of climate can promote vegetation growth [1,69,75]. However, after eliminating the effects of precipitation and solar radiation, this article found that the correlation between NPP and temperature rapidly decreased, and the area that reached a significant level also rapidly decreased. Some areas also showed a negative correlation, mainly distributed on cultivated land on both sides of the Yangtze and Huaihe Rivers. Rice is planted on both sides of the Yangtze River, and wheat is planted on both sides of the Huaihe River. High temperatures can inhibit the growth of two types of crops, which rely more on water and light hours. Du et al. [55] reached a similar conclusion when studying the partial correlation analysis between NDVI and temperature, which is that there is a negative correlation in the western and southern parts of Anhui Province. After eliminating the effects of temperature and solar radiation, the correlation between precipitation and NPP has also weakened, with negative correlations appearing on both sides of the Yangtze River and around lakes where soil moisture is abundant and excessive precipitation can inhibit vegetation growth. After eliminating the effects of temperature and precipitation, the correlation between solar radiation and NPP slightly decreased. Similar to the results of simple correlation analysis, except for water, all other regions showed a positive correlation. The explanation is that any unilateral change in climate factors is not sufficient to cause significant changes in NPP of large-scale pixel groups, but rather the result of the coupling effect of heat, water, and energy.

The response of NPP to climate factors varies among different land cover types. The partial correlation coefficients between cultivated land and temperature, precipitation, and solar radiation are 0.03, 0.29, and 0.46, respectively, with some showing a negative correlation. This may be because some crops in Anhui Province that are suitable for low-temperature growth are limited in carbon accumulation during high temperatures, while sufficient rainfall and longer sunshine hours result in more carbon accumulation. Especially in the Jianghuai region, it is greatly affected by precipitation, due to the fact that the vegetation in this area is mainly dry land. In forested areas, the partial correlation coefficients of temperature, precipitation, and solar radiation are 0.01, 0.21, and 0.55, respectively. The forest land in Anhui Province is located in relatively high altitude mountainous areas, with a large distribution of warm and cool vegetation and strong drought resistance. The longer the sunshine hours, the more favorable it is for carbon accumulation. This is similar to Du et al. [56], who mentioned in his article that the partial correlation coefficient between forest NPP in Anhui Province and temperature and precipitation is around 0, while the partial correlation coefficient with solar radiation is around 0.45. The partial correlation coefficients between grassland and temperature, precipitation, and solar radiation are 0.03, 0.29, and 0.48, respectively. The partial correlation coefficients between cities and temperature, precipitation, and solar radiation are 0.03, 0.31, and 0.42, respectively Relatively speaking, vegetation in grasslands and cities has a higher dependence on precipitation than other land cover types. Increasing precipitation can promote vegetation growth and increase the annual NPP of grasslands by increasing soil moisture in the region [76]. The more precipitation, the longer the sunshine hours, which are beneficial for carbon accumulation. Among other land cover types, the proportion of correlation with NPP reaching a significant level is relatively small. Overall, through partial correlation analysis, it was found that solar radiation has the greatest impact on the NPP of Anhui Province, followed by precipitation, and the least impact on temperature.

(a) partial correlation analysis between NPP and temperature.

(b) partial correlation analysis between NPP and precipitation.

(c) partial correlation analysis between NPP and solar radiation.

Fig 11. Spatial distribution of the partial correlation analysis between NPP and meteorological factors.

**Line 428 to 444:**

Land use and land cover change (LUCC) will directly affect the types, structures, and functions of ecosystems, and then have important impact on NPP. The most important land cover changes in Anhui Province from 2001 to 2020 was cultivated land to artificial surfaces, as well as the transformation between cultivated land and forest (See Table 5). Among all land cover types, the average annual NPP decreased most obviously, followed by bare land, and the average annual NPP increased for other land cover types. In order to understand the impact of land use change on NPP, we focused on studying the transformation from various land use types to artificial surfaces [28]. The area of artificial surfaces has increased by 4555.71 km^2^ in the past two decades. From Table 5, it can be seen that a total of 7377.80 km^2^ of cultivated land was converted into artificial surfaces, 406.27 km^2^ of forest has been converted into artificial surfaces, and 122.69 km^2^ of grassland has been converted into artificial surfaces. The unit area NPP of cultivated land, forest, and grassland are larger than that of artificial surfaces, so this transformation will cause losses in NPP. At the same time, water, wetlands, and bare land are converted into artificial surfaces, and their unit area NPP are smaller than that of artificial surfaces, which can lead to an increase in NPP. The urban expansion in Anhui Province is mainly derived from the conversion of cultivated land, so the urban expansion in Anhui Province has led to a decrease in NPP. The conclusion of Wang et al. [33] also verified the significant negative effect of urbanization on the productivity of vegetation cover system in Anhui province. Urban sprawl offsets the increase in NPP caused by climate change. The results of Yang et al. also indicate that the changes of NPP in the Yangtze River Basin are jointly influenced by LUCC changes and climate changes [60,75].

**Added references:**

33.	Wang S, Wei G, Gao M, Fan Y. Investigating the mechanism of urbanization on the net primary productivity of vegetation in the Yangtze River Economic Belt: a comprehensive analysis from global and local effects. Frontiers in Ecology and Evolution. 2023;11: 1231487-1231501. doi: 10.3389/fevo.2023.1231487.

50.	Ma R, Xia C, Liu Y, Wang Y, Zhang J, Shen X, et al. Spatiotemporal Change of Net Primary Productivity and Its Response to Climate Change in Temperate Grasslands of China. Frontiers in Plant Science. 2022;13:899800-899811. doi: 10.3389/fpls.2022.899800.

55.	Du Z, Zhao J, Liu X, Wu Z, Zhang HJES, Research P. Recent asymmetric warming trends of daytime versus nighttime and their linkages with vegetation greenness in temperate China. Environmental Science and Pollution Research. 2019;26:35717-35727. doi: 10.1007/s11356-019-06440-z.

56.	Du Z, Liu X, Wu Z, Zhang H, Zhao J. Responses of Forest Net Primary Productivity to Climatic Factors in China during 1982–2015. Plants. 2022;11:2932-2949. doi: 10.3390/plants11212932.

60.	Yang H, Zhong X, Deng S, Xu H. Assessment of the impact of LUCC on NPP and its influencing factors in the Yangtze River basin, China. CATENA. 2021;206: 105542-105554. doi: 10.1016/j.catena.2021.105542.

69.	Fang X, Zhang C, Wang Q, Chen X, Ding J, Karamage F. Isolating and Quantifying the Effects of Climate and CO2 Changes (1980–2014) on the Net Primary Productivity in Arid and Semiarid China. Forests. 2017;8:60-79. doi: 10.3390/f8030060.

70.	Fu Y, Lu X, Zhao Y, Zeng X, Xia L. Assessment Impacts of Weather and Land Use/Land Cover (LULC) Change on Urban Vegetation Net Primary Productivity (NPP): A Case Study in Guangzhou, China. Remote Sensing. 2013;5:4125-4144. doi: 10.3390/rs5084125. PubMed PMID: doi:10.3390/rs5084125.

70.	Pei F, Li X, Liu X, Lao C, Xia G. Exploring the response of net primary productivity variations to urban expansion and climate change: A scenario analysis for Guangdong Province in China. Journal of Environmental Management. 2015;150:92-102. doi: 10.1016/j.jenvman.2014.11.002.

72.	Shen M, Cong N, Cao R. Temperature sensitivity as an explanation of the latitudinal pattern of green-up date trend in Northern Hemisphere vegetation during 1982-2008. International Journal of Climatology. 2014;35:3707-3712. doi: 10.1002/joc.4227.

73.	Xiong L, Bai X, Zhao C, Li Y, Tan Q, Yun L, et al. High‐Resolution Data Sets for Global Carbonate and Silicate Rock Weathering Carbon Sinks and Their Change Trends. Earth's Future. 2022;10:e2022EF002746- e2022EF002763. doi: 10.1029/2022EF002746.

75.	Yang H, Hu D, Xu H, Zhong X. Assessing the spatiotemporal variation of NPP and its response to driving factors in Anhui province, China. Environmental Science and Pollution Research. 2020;27: 14915-14932. doi: 10.1007/s11356-020-08006-w.

76.	Guo D, Song X, Hu R, Cai S, Zhu X, Hao Y. Grassland type-dependent spatiotemporal characteristics of productivity in Inner Mongolia and its response to climate factors. Science of The Total Environment. 2021;775:145644-53. doi: 10.1016/j.scitotenv.2021.145644.

Is the land use data dynamic? How did the author consider Land Cover Change as an independent variable?

**Response:** Thank you for the positive and valuable comments. Thanks to the reviewer for highlighting our unique aspects. Land use data is dynamic. Therefore, this article calculates the NPP of land use types that have not changed based on the average NPP from 2001 to 2020. The unit area NPP of forest (603.17 gC·(m^2^ ·a) ^-1^) is the highest, followed by grassland (487.06 gC·(m^2^ ·a) ^-1^), followed by cultivated land (462.71 gC·(m^2^ ·a) ^-1^) , artificial surfaces (431.68 gC·(m^2^ ·a) ^-1^) , wetland (332.32 gC·(m^2^ ·a) ^-1^) and bareland (250.57 gC·(m^2^ ·a) ^-1^) , The unit area NPP of water (217.64 gC·(m^2^ ·a) ^-1^) is the lowest . Changes in land use types can lead to changes in NPP, such as the conversion from forest land to other land use types, which can result in losses of NPP. The transformation from water bodies to other land use types will result in an increase in NPP.

**After revision:**

**Line 428 to 444:**

Land use and land cover change (LUCC) will directly affect the types, structures, and functions of ecosystems, and then have important impact on NPP. The most important land cover changes in Anhui Province from 2001 to 2020 was cultivated land to artificial surfaces, as well as the transformation between cultivated land and forest (See Table 5). Among all land cover types, the average annual NPP decreased most obviously, followed by bare land, and the average annual NPP increased for other land cover types. In order to understand the impact of land use change on NPP, we focused on studying the transformation from various land use types to artificial surfaces [28]. The area of artificial surfaces has increased by 4555.71 km^2^ in the past two decades. From Table 5, it can be seen that a total of 7377.80 km^2^ of cultivated land was converted into artificial surfaces, 406.27 km^2^ of forest has been converted into artificial surfaces, and 122.69 km^2^ of grassland has been converted into artificial surfaces. The unit area NPP of cultivated land, forest, and grassland are larger than that of artificial surfaces, so this transformation will cause losses in NPP. At the same time, water, wetlands, and bare land are converted into artificial surfaces, and their unit area NPP are smaller than that of artificial surfaces, which can lead to an increase in NPP. The urban expansion in Anhui Province is mainly derived from the conversion of cultivated land, so the urban expansion in Anhui Province has led to a decrease in NPP. The conclusion of Wang et al. [33] also verified the significant negative effect of urbanization on the productivity of vegetation cover system in Anhui province. Urban sprawl offsets the increase in NPP caused by climate change. The results of Yang et al. also indicate that the changes of NPP in the Yangtze River Basin are jointly influenced by LUCC changes and climate changes [60,75].

**Added references:**

28.	Li J, Wang Z, Lai C, Wu X, Zeng Z, Chen X, et al. Response of net primary production to land use and land cover change in mainland China since the late 1980s. The Science of the total environment. 2018;639:237-247. doi: 10.1016/j.scitotenv.2018.05.155.

The research methods section should provide specific methods for "Climate change and Land Use/Land Cover Change leading to spatial shifts in Net Primary Productivity."

**Response:** Thank you for the positive and valuable comments. We have added descriptions of methods for trend analysis, simple correlation analysis, and partial correlation analysis.

**After revision:**

**Lines 157-187:**

**Trend analysis**

The trend line method of univariate linear regression can reflect the trend characteristics of pixel value changes in different periods by analyzing the size of pixel values [48]. This article uses this method to analyze the trend of simulated NPP changes in Anhui Province from 2001 to 2020, and the calculation formula is as follows [49-50]:

$slope={(n*\sum_{i=1}^{n} (i*Y_{i})-\sum_{i=1}^{n} i*\sum_{i=1}^{n} Y_{i})}/{(n*\sum_{i=1}^{n} i^{2}-({\sum_{i=1}^{n} i)}^{2})}$	(2)

Slope is the trend of NPP change, i is the annual variable, n is the number of years, and Y_i_ is the NPP value in the i-th year. When slope is greater than 0, NPP increases, otherwise it decreases.

**Simple correlation coefficient analysis**

Simple correlation coefficient is the calculation of the correlation between simulated NPP values and climate factors (temperature, precipitation, and solar radiation). The calculation formula is as follows [45,51,52]:

$r_{xy}={\sum_{i=1}^{n} [\left( x_{i}-\bar{x} \right)*(y_{i}-\bar{y})]}/{\sqrt{\sum_{i=1}^{n} \left( x_{i}-\bar{x} \right)^{2}*\sum_{i=1}^{n} {(y_{i}-\bar{y})}^{2}}}$	(3)

r_xy_ is the simple correlation coefficient between x and y, x_i_ is the NPP value for the i-th year or month, y_i_ is the correlation factor value for the corresponding year or month, $\bar{x}\mathrm{and}\bar{y}$ are the average NPP value and the corresponding correlation factor average, respectively, and i is the annual variable.

**Partial correlation analysis**

Coefficient of partial correlation is determined based on the simple correlation coefficient. The partial correlation coefficient can be used to analyze the correlation between multiple variables, that is, to calculate the correlation coefficient between two variables after fixing one variable, which can be used in many cases to assess a said relationship [53-54]. This article calculates the partial correlation coefficient between NPP and temperature by fixing solar radiation and precipitation. Similarly, when calculating the partial correlation coefficient between NPP and precipitation, two variables, temperature and solar radiation, were fixed. The calculation of the partial correlation coefficient between NPP and solar radiation involves two variables: fixed temperature and precipitation [55-56]. The calculation formula is as follows [50]:

$r_{y1,2}={(r_{y1}-r_{y2}*r_{12})}/{\sqrt{\left( 1-r_{y2}^{2} \right)*(1-r_{12}^{2})}}$	(4)

r_y1,2_ represents the partial correlation coefficient between variable y and variable x1 after fixed variable x2. r_y1_, r_y2_, and r_12_ represent the correlation coefficients between y and x1, y and x2, and x1 and x2, respectively.

The trend analysis, simple correlation coefficient and partial correlation analysis were performed in the R environment with package of ‘stats’ (version 3.4.4) and ‘ppcor’ (version 1.1), respectively.

**Added references:**

48.	Stow D, Daeschner S, Hope A, Douglas D, Petersen A, Myneni R, et al. Variability of the Seasonally Integrated Normalized Difference Vegetation Index Across the North Slope of Alaska in the 1990s. International Journal of Remote Sensing. 2003;24:1111-1117. doi: 10.1080/0143116021000020144.

49.	Zhuang Q, Shao Z, Li D, Huang X, Cai B, Altan O, et al. Unequal weakening of urbanization and soil salinization on vegetation production capacity. Geoderma. 2022;411:115712-115723. doi: 10.1016/j.geoderma.2022.115712.

50.	Ma R, Xia C, Liu Y, Wang Y, Zhang J, Shen X, et al. Spatiotemporal Change of Net Primary Productivity and Its Response to Climate Change in Temperate Grasslands of China. Frontiers in Plant Science. 2022;13:899800-899811. doi: 10.3389/fpls.2022.899800.

51.	Shen X, Liu B, Jiang M, Wang Y, Wang L, Zhang J, et al. Spatiotemporal Change of Marsh Vegetation and Its Response to Climate Change in China From 2000 to 2019. Journal of Geophysical Research: Biogeosciences. 2021;126: e2020JG00615401- e2020JG00615413. doi: 10.1029/2020JG006154.

52.	Shen X, Liu Y, Zhang J, Wang Y, Ma R, Liu B, et al. Asymmetric Impacts of Diurnal Warming on Vegetation Carbon Sequestration of Marshes in the Qinghai Tibet Plateau. Global Biogeochemical Cycles. 2022;36: e2022GB007396- e2022GB007409. doi: 10.1029/2022GB007396.

53.	Shen M, Piao S, Chen X, An S, Fu Y, Wang S, et al. Strong impacts of daily minimum temperature on the green-up date and summer greenness of the Tibetan Plateau. Global Change Biology. 2016;22:3057-3066. doi: 10.1111/gcb.13301.

54.	Tan J, Piao S, Chen A, Zeng Z, Ciais P, Janssens I, et al. Seasonally different response of photosynthetic activity to daytime and night-time warming in the Northern Hemisphere. Global Change Biology. 2014;21:377–387. doi: 10.1111/gcb.12724.

Figures 1, 5, and 9 in the text should be numbered, and the p-value should be provided in Figure 5.

**Response:** Thank you for the positive and valuable comments. We have added number in Figures 1, 5, and 9, and added P-values in Figure 5.

**After revision:**

**Lines 102:**

Fig 1. Geographical location of the study area.

**Lines 228:**

Fig 5. Variation trend of NPP in different land use types.

**Lines 300:**

Fig 9. Relationship between temperature, precipitation and NPP.

**Other comments：**

1. Please ensure that your manuscript meets PLOS ONE's style requirements, including those for file naming. The PLOS ONE style templates can be found at

https://journals.plos.org/plosone/s/file?id=wjVg/PLOSOne_formatting_sample_main_body.pdf and

https://journals.plos.org/plosone/s/file?id=ba62/PLOSOne_formatting_sample_title_authors_affiliations.pdf

**Response:** Thank you for the positive and valuable comments. The whole manuscript has been edited according to your templates.

2. In your Methods section, please provide additional information regarding the permits you obtained for the work. Please ensure you have included the full name of the authority that approved the field site access and, if no permits were required, a brief statement explaining why.

**Response:** Thank you for the positive and valuable comments. Thanks to the reviewer for highlighting our unique aspects. In the Methods section, we have added data sources, Data Availability Statement and expressed our gratitude in the acknowledgments section.

**After revision:**

**Line 106 to 131:**

**Table 1. Data sources.**

| **Data** | **Spatial resolution** | **Sources** |
| --- | --- | --- |
| Vector boundary data of China and Anhui Province | vector | https://www.webmap.cn/commres.do?method=result100W |
| MOD13Q1 NDVI | 250m×250m | https://ladsweb.modaps.eosdis.nasa.gov/search/ |
| monthly mean temperature | - | https://www.resdc.cn/data.aspx?DATAID=349, GST_ave |
| monthly total solar radiation | - | https://www.resdc.cn/data.aspx?DATAID=349, SSD |
| monthly total precipitation | - | https://www.resdc.cn/data.aspx?DATAID=349, PRE |
| Vegetation type | 1km×1km | [https://www.plantplus.cn/doi/10.12282/plantdata.0155](https://www.plantplus.cn/doi/10.12282/plantdata.0155%20) |
| land-use and land-cover | 30m×30m | https://www.webmap.cn/mapDataAction.do?method=globalLandCover |

There are five main types of data.

(1) Vector boundary data. One type is the vector map of the entire region of China, and the other is the administrative boundary map of Anhui Province. These data are all sourced from national catalogue service for geographic information (https://www.webmap.cn/commres.do?method=result100W).

(2) NDVI time series data (2001-2020). It is a grid file, derived from MODIS data, using the NASA website MOD13Q1 series products (https://search.earthdata.nasa.gov), with 16 days as the cycle of NDVI data of 250 - meter resolution. It is obtained by batch clipping, trans- projection, and maximum value synthesis. The NDVI data of 12 months of a year were combined to form the average annual NDVI distribution map.

(3) Meteorological data. The climate dataset was derived from 77 climatological stations (Fig 1(b)) in Anhui province, including three types of data from 2001 to 2020: temperature, precipitation, and solar radiation, and were provided by Resource and Environmental Science Data Platform (https://www.resdc.cn/data.aspx?DATAID=349). To ensure the quality of data, we delete the suspicious and missing records. Since many researches have demonstrated that the Kriging method is an effective interpolation method, having been widely used for regionalizing various variables at different scales [44-45], these climatological data were interpolated through the Kriging method, to produce raster images. All data were resampled at a resolution of 250 m× 250 m and repeatedly projected for WGS_1984_Albers. Time range consistent with NDVI.

(4) Vegetation type map is a raster file to determine the spatial distribution of various vegetation types. Based on 1:1 million Chinese vegetation type map compiled by the Vegetation Map Editing Committee of the Chinese Academy of Sciences in 2001[46], all vegetation types were re-screened, merged, and coded. The main vegetation types in Anhui province are needle leaved forest, broadleaved forest, bush, grassland, swamp, farmland, and water body.

(5) land-use and land-cover (LULC). The remote sensing data with a resolution of 30m*30m was provided by national catalogue service for geographic information (https://www.webmap.cn/mapDataAction.do?method=globalLandCover). We resampled it to a resolution of 250 meters using the nearest neighbor method based on ArcGIS 10.8 [45]. Land use types include divided into Cultivated Land, Forest, Grassland, Wetland, Water, Artificial Surfaces and Bare land.

**Line 520 to 529:**

**Data Availability Statement:** The minimum dataset required to replicate our research results (mainly includes MOD13Q1 NDVI, Meteorological data, Vegetation type, LULC data and Vector boundary data) is located in the manuscript. In addition, the MOD13Q1 NDVI, Meteorological data, Vegetation type, LULC data and Vector boundary data is third-party data, and the authors had no special access privileges to the data and that other researchers will be able to access the data in the same manner as the authors. They can be obtained in the following ways: 1. MOD13Q1 NDVI product is available from https://ladsweb.modaps.eosdis.nasa.gov. 2. Meteorological data is available from https://www.resdc.cn/. 3. Vegetation type is available from Vegetation map of the People’s Republic of China (1:1 million) ，Xinshi Zhang,Vegetation Map Editing Committee of the Chinese Academy of Sciences (https://www.plantplus.cn/doi/10.12282/plantdata.0155), doi:10.12282/plantdata. 4. LULC product is available from https://www.webmap.cn/mapDataAction.do?method=globalLandCover. 5. Vector boundary data is available from https://www.webmap.cn/.

**Acknowledgments:** Thanks to the Plant Science Data Center of Chinese Academy of Sciences (https://www.plantplus.cn) and Resource and Environmental Science Data Platform (https://www.resdc.cn/) for providing data support. Authors appreciate the reviewers for their invaluable comments which have led to significant improvement in the paper.

**Added references:**

46.	Zhang X, cartographer Vegetation map of the People's Republic of China (1:1 000 000). Beijing, China: Geology Press; 2007. DOI:10.12282/plantdata.0155, CSTR:34735.11.PLANTDATA.0155.

3. We note that the grant information you provided in the ‘Funding Information’ and ‘Financial Disclosure’ sections do not match.

When you resubmit, please ensure that you provide the correct grant numbers for the awards you received for your study in the ‘Funding Information’ section.

**Response:** Thank you for the positive and valuable comments. Thanks to the reviewer for highlighting our unique aspects. You are right, funding information should not appear in the Acknowledgments section or other areas of your manuscript. Funding Information with the correct grant numbers for the awards are provided in the Funding Statement section of the online submission form.

**After revision:**

4. Thank you for stating the following in the Acknowledgments Section of your manuscript:

“The authors received no specific funding for this work.”

We note that you have provided funding information that is not currently declared in your Funding Statement. However, funding information should not appear in the Acknowledgments section or other areas of your manuscript. We will only publish funding information present in the Funding Statement section of the online submission form.

Please remove any funding-related text from the manuscript and let us know how you would like to update your Funding Statement. Currently, your Funding Statement reads as follows:

"The paper was partially supported by National Natural Science Foundation of China (42271301), Anhui University Excellent Research and Innovation Project (No. 2022AH010094). Authors appreciate the reviewers for their invaluable comments which have led to significant improvement in the paper."

Please include your amended statements within your cover letter; we will change the online submission form on your behalf.

**Response:** Thank you for the positive and valuable comments. You are right, funding information should not appear in the Acknowledgments section or other areas of your manuscript. Funding Information with the correct grant numbers for the awards are provided in the Funding Statement section of the online submission form.

**After revision:**

**Line 520 to 529:**

**Data Availability Statement:** The minimum dataset required to replicate our research results (mainly includes MOD13Q1 NDVI, Meteorological data, Vegetation type, LULC data and Vector boundary data) is located in the manuscript. In addition, the MOD13Q1 NDVI, Meteorological data, Vegetation type, LULC data and Vector boundary data is third-party data, and the authors had no special access privileges to the data and that other researchers will be able to access the data in the same manner as the authors. They can be obtained in the following ways: 1. MOD13Q1 NDVI product is available from https://ladsweb.modaps.eosdis.nasa.gov. 2. Meteorological data is available from https://www.resdc.cn/. 3. Vegetation type is available from Vegetation map of the People’s Republic of China (1:1 million) ，Xinshi Zhang,Vegetation Map Editing Committee of the Chinese Academy of Sciences (https://www.plantplus.cn/doi/10.12282/plantdata.0155), doi:10.12282/plantdata. 4. LULC product is available from https://www.webmap.cn/mapDataAction.do?method=globalLandCover. 5. Vector boundary data is available from https://www.webmap.cn/.

**Acknowledgments:** Thanks to the Plant Science Data Center of Chinese Academy of Sciences (https://www.plantplus.cn) and Resource and Environmental Science Data Platform (https://www.resdc.cn/) for providing data support. Authors appreciate the reviewers for their invaluable comments which have led to significant improvement in the paper.

5. Please provide a complete Data Availability Statement in the submission form, ensuring you include all necessary access information or a reason for why you are unable to make your data freely accessible. If your research concerns only data provided within your submission, please write "All data are in the manuscript and/or supporting information files" as your Data Availability Statement.

**Response:** Thank you for the positive and valuable comments. You are right, Data Availability Statement “The data used and/or analyzed during the current study are in the manuscript and/or available from the corresponding author on reasonable request." has been added in the paper.

**After revision:**

**Line 106:**

**Table 1. Data sources.**

| **Data** | **Spatial resolution** | **Sources** |
| --- | --- | --- |
| Vector boundary data of China and Anhui Province | vector | https://www.webmap.cn/commres.do?method=result100W |
| MOD13Q1 NDVI | 250m×250m | https://ladsweb.modaps.eosdis.nasa.gov/search/ |
| monthly mean temperature | - | https://www.resdc.cn/data.aspx?DATAID=349, GST_ave |
| monthly total solar radiation | - | https://www.resdc.cn/data.aspx?DATAID=349, SSD |
| monthly total precipitation | - | https://www.resdc.cn/data.aspx?DATAID=349, PRE |
| Vegetation type | 1km×1km | [https://www.plantplus.cn/doi/10.12282/plantdata.0155](https://www.plantplus.cn/doi/10.12282/plantdata.0155%20) |
| land-use and land-cover | 30m×30m | https://www.webmap.cn/mapDataAction.do?method=globalLandCover |

**Line 520 to 529:**

**Data Availability Statement:** The minimum dataset required to replicate our research results (mainly includes MOD13Q1 NDVI, Meteorological data, Vegetation type, LULC data and Vector boundary data) is located in the manuscript. In addition, the MOD13Q1 NDVI, Meteorological data, Vegetation type, LULC data and Vector boundary data is third-party data, and the authors had no special access privileges to the data and that other researchers will be able to access the data in the same manner as the authors. They can be obtained in the following ways: 1. MOD13Q1 NDVI product is available from https://ladsweb.modaps.eosdis.nasa.gov. 2. Meteorological data is available from https://www.resdc.cn/. 3. Vegetation type is available from Vegetation map of the People’s Republic of China (1:1 million) ，Xinshi Zhang,Vegetation Map Editing Committee of the Chinese Academy of Sciences (https://www.plantplus.cn/doi/10.12282/plantdata.0155), doi:10.12282/plantdata. 4. LULC product is available from https://www.webmap.cn/mapDataAction.do?method=globalLandCover. 5. Vector boundary data is available from https://www.webmap.cn/.

**Acknowledgments:** Thanks to the Plant Science Data Center of Chinese Academy of Sciences (https://www.plantplus.cn) and Resource and Environmental Science Data Platform (https://www.resdc.cn/) for providing data support. Authors appreciate the reviewers for their invaluable comments which have led to significant improvement in the paper.

6. We note that [Figures 1, 7, 8a, 8b, 10a, 10b and 10c] in your submission contain [map/satellite] images which may be copyrighted. All PLOS content is published under the Creative Commons Attribution License (CC BY 4.0), which means that the manuscript, images, and Supporting Information files will be freely available online, and any third party is permitted to access, download, copy, distribute, and use these materials in any way, even commercially, with proper attribution. For these reasons, we cannot publish previously copyrighted maps or satellite images created using proprietary data, such as Google software (Google Maps, Street View, and Earth). For more information, see our copyright guidelines: http://journals.plos.org/plosone/s/licenses-and-copyright.

We require you to either (1) present written permission from the copyright holder to publish these figures specifically under the CC BY 4.0 license, or (2) remove the figures from your submission:

a. You may seek permission from the original copyright holder of Figures 1, 7, 8a, 8b, 10a, 10b and 10c to publish the content specifically under the CC BY 4.0 license.

We recommend that you contact the original copyright holder with the Content Permission Form (http://journals.plos.org/plosone/s/file?id=7c09/content-permission-form.pdf) and the following text:

“I request permission for the open-access journal PLOS ONE to publish XXX under the Creative Commons Attribution License (CCAL) CC BY 4.0 (http://creativecommons.org/licenses/by/4.0/). Please be aware that this license allows unrestricted use and distribution, even commercially, by third parties. Please reply and provide explicit written permission to publish XXX under a CC BY license and complete the attached form.”

Please upload the completed Content Permission Form or other proof of granted permissions as an "Other" file with your submission.

In the figure caption of the copyrighted figure, please include the following text: “Reprinted from [ref] under a CC BY license, with permission from [name of publisher], original copyright [original copyright year].”

b. If you are unable to obtain permission from the original copyright holder to publish these figures under the CC BY 4.0 license or if the copyright holder’s requirements are incompatible with the CC BY 4.0 license, please either i) remove the figure or ii) supply a replacement figure that complies with the CC BY 4.0 license. Please check copyright information on all replacement figures and update the figure caption with source information. If applicable, please specify in the figure caption text when a figure is similar but not identical to the original image and is therefore for illustrative purposes only.

The following resources for replacing copyrighted map figures may be helpful:

USGS National Map Viewer (public domain): http://viewer.nationalmap.gov/viewer/

The Gateway to Astronaut Photography of Earth (public domain): http://eol.jsc.nasa.gov/sseop/clickmap/

Maps at the CIA (public domain): https://www.cia.gov/library/publications/the-world-factbook/index.html and https://www.cia.gov/library/publications/cia-maps-publications/index.html

NASA Earth Observatory (public domain): http://earthobservatory.nasa.gov/

Landsat: http://landsat.visibleearth.nasa.gov/

USGS EROS (Earth Resources Observatory and Science (EROS) Center) (public domain): http://eros.usgs.gov/#

Natural Earth (public domain): http://www.naturalearthdata.com/

**Response:** Thank you for the positive and valuable comments. Figure 1 is a graph generated using administrative boundary data and digital elevation models. Figure 7 shows the results obtained by inputting MODIS NDVI, meteorological data, and vegetation types into the CASA model. Figures 8a, 8b, 10a, 10b, 10c, 11a, 11b, and 11c show the trend analysis, simple correlation analysis, and partial correlation analysis based on the results of Figure 7. All graphs were exported in ArcGIS 10.8. The minimum dataset required to replicate our research results is located in the manuscript.

**After revision:**

**Line 94-100:**

Anhui Province is located in East China, the Yangtze River Delta region, the terrain from north to south by plain, hills, mountains, within the Yangtze River, Huaihe River and Chaohu Lake, can be divided into three areas north of the Huai River, Jianghuai area and south of the Yangtze River (See Fig 1, Digital Elevation Model is downloaded for free from Geospatial Data Cloud, https://www.gscloud.cn/sources/accessdata/310?pid=302). Anhui Province is a transitional region between warm temperate zone and subtropical zone. North of Huaihe River is a warm temperate semi-humid monsoon climate, south of Huaihe River is a sub-hot humid monsoon climate. The average annual temperature is 14-17 ℃, and the average annual precipitation is 773-1670 mm.

**Line 105-131:**

**Table 1. Data sources.**

| **Data** | **Spatial resolution** | **Sources** |
| --- | --- | --- |
| Vector boundary data of China and Anhui Province | vector | https://www.webmap.cn/commres.do?method=result100W |
| MOD13Q1 NDVI | 250m×250m | https://ladsweb.modaps.eosdis.nasa.gov/search/ |
| monthly mean temperature | - | https://www.resdc.cn/data.aspx?DATAID=349, GST_ave |
| monthly total solar radiation | - | https://www.resdc.cn/data.aspx?DATAID=349, SSD |
| monthly total precipitation | - | https://www.resdc.cn/data.aspx?DATAID=349, PRE |
| Vegetation type | 1km×1km | [https://www.plantplus.cn/doi/10.12282/plantdata.0155](https://www.plantplus.cn/doi/10.12282/plantdata.0155%20) |
| land-use and land-cover | 30m×30m | https://www.webmap.cn/mapDataAction.do?method=globalLandCover |

There are five main types of data.

(1) Vector boundary data. One type is the vector map of the entire region of China, and the other is the administrative boundary map of Anhui Province. These data are all sourced from national catalogue service for geographic information (https://www.webmap.cn/commres.do?method=result100W).

(2) NDVI time series data (2001-2020). It is a grid file, derived from MODIS data, using the NASA website MOD13Q1 series products (https://search.earthdata.nasa.gov), with 16 days as the cycle of NDVI data of 250 - meter resolution. It is obtained by batch clipping, trans- projection, and maximum value synthesis. The NDVI data of 12 months of a year were combined to form the average annual NDVI distribution map.

(3) Meteorological data. The climate dataset was derived from 77 climatological stations (Fig 1(b)) in Anhui province, including three types of data from 2001 to 2020: temperature, precipitation, and solar radiation, and were provided by Resource and Environmental Science Data Platform (https://www.resdc.cn/data.aspx?DATAID=349). To ensure the quality of data, we delete the suspicious and missing records. Since many researches have demonstrated that the Kriging method is an effective interpolation method, having been widely used for regionalizing various variables at different scales [44-45], these climatological data were interpolated through the Kriging method, to produce raster images. All data were resampled at a resolution of 250 m× 250 m and repeatedly projected for WGS_1984_Albers. Time range consistent with NDVI.

(4) Vegetation type map is a raster file to determine the spatial distribution of various vegetation types. Based on 1:1 million Chinese vegetation type map compiled by the Vegetation Map Editing Committee of the Chinese Academy of Sciences in 2001[46], all vegetation types were re-screened, merged, and coded. The main vegetation types in Anhui province are needle leaved forest, broadleaved forest, bush, grassland, swamp, farmland, and water body.

(5) land-use and land-cover (LULC). The remote sensing data with a resolution of 30m*30m was provided by national catalogue service for geographic information (https://www.webmap.cn/mapDataAction.do?method=globalLandCover). We resampled it to a resolution of 250 meters using the nearest neighbor method based on ArcGIS 10.8 [45]. Land use types include divided into Cultivated Land, Forest, Grassland, Wetland, Water, Artificial Surfaces and Bare land.

**Line 520 to 529:**

**Data Availability Statement:** The minimum dataset required to replicate our research results (mainly includes MOD13Q1 NDVI, Meteorological data, Vegetation type, LULC data and Vector boundary data) is located in the manuscript. In addition, the MOD13Q1 NDVI, Meteorological data, Vegetation type, LULC data and Vector boundary data is third-party data, and the authors had no special access privileges to the data and that other researchers will be able to access the data in the same manner as the authors. They can be obtained in the following ways: 1. MOD13Q1 NDVI product is available from https://ladsweb.modaps.eosdis.nasa.gov. 2. Meteorological data is available from https://www.resdc.cn/. 3. Vegetation type is available from Vegetation map of the People’s Republic of China (1:1 million) ，Xinshi Zhang,Vegetation Map Editing Committee of the Chinese Academy of Sciences (https://www.plantplus.cn/doi/10.12282/plantdata.0155), doi:10.12282/plantdata. 4. LULC product is available from https://www.webmap.cn/mapDataAction.do?method=globalLandCover. 5. Vector boundary data is available from https://www.webmap.cn/.

**Acknowledgments:** Thanks to the Plant Science Data Center of Chinese Academy of Sciences (https://www.plantplus.cn) and Resource and Environmental Science Data Platform (https://www.resdc.cn/) for providing data support. Authors appreciate the reviewers for their invaluable comments which have led to significant improvement in the paper.

Finally, the whole text and figures has been revised and checked by third person to assure the quality of the English.

Thanks again for your valuable comments. Stay healthy and best wishes. We really appreciated your help. If you have any other concern, please don’t hesitate to let us know. I look forward to hearing from you very soon.

Warmest Regards,

Huan Tang & Jing Yuan
